# Abnormalities in lysine degradation are involved in early cardiomyocyte hypertrophy development in pressure-overloaded rats

**DOI:** 10.1186/s12872-021-02209-w

**Published:** 2021-08-21

**Authors:** Jialing Liu, Junhao Hu, Lanlan Tan, Qi Zhou, Xiaojing Wu

**Affiliations:** 1 Cardiovascular Department of the Second Affiliated Hospital of Chongqing Medical University, NO.74 Linjiang Road, Yuzhong District, Chongqing, 400010 People’s Republic of China; 2 Cardiovascular Department of Shenzhen University General Hospital and Shenzhen University Clinical Medical Academy, NO.1098 Xueyuan Street, Nanshan District, Shenzhen, 518060 People’s Republic of China

**Keywords:** Cardiac hypertrophy, Pressure overload, Lysine degradation, Metabolomics, Nonenergetic metabolism

## Abstract

**Background:**

Cardiomyocyte metabolism changes before cardiac remodeling, but its role in early cardiac hypertrophy detection remains unclear. This study investigated early changes in plasma metabolomics in a pressure-overload cardiac hypertrophy model induced by transverse aortic constriction (TAC).

**Methods:**

The TAC model was constructed by partly ligating the aortic arch. Twelve Sprague–Dawley rats were randomly divided into the TAC group (n = 6) and sham group (n = 6). Three weeks after surgery, cardiac echocardiography was performed to assess cardiac remodeling and function. Hematoxylin/eosin (HE), Masson, and wheat germ agglutinin (WGA) stains were used to observe pathological changes. Plasma metabolites were detected by UPLC-QTOFMS and Q-TOFMS. Specific metabolites were screened by orthogonal partial least squares discriminant analysis (OPLS-DA). Metabolic pathways were characterized by Kyoto Encyclopedia of Genes and Genomes (KEGG) analysis, and the predictive value of the screened metabolites was analyzed by receiver operating characteristic (ROC) curve analysis.

**Results:**

Three weeks after surgery, the TAC and sham groups had similar left heart function and interventricular septum and diastolic left ventricular posterior wall thicknesses. However, on pathological examination, the cross-sectional area of cardiomyocytes and myocardial fibrosis severity were significantly elevated in TAC rats. OPLS-DA showed different metabolic patterns between the TAC and sham groups. Based on the criteria VIP > 1 and P < 0.05, 13 metabolites were screened out. KEGG analysis identified disrupted lysine degradation through the related metabolites 5-aminopentanoic acid, N6-acetyl-l-lysine, and l-lysine, with areas under the ROC curve (AUCs) of 0.917, 0.889, and 0.806, respectively, for predicting compensated cardiomyocyte hypertrophy.

**Conclusion:**

Disruption of lysine degradation might be involved in early cardiac hypertrophy development, and related metabolites might be potential predictive and interventional targets for subclinical cardiomyocyte hypertrophy.

## Background

Cardiac hypertrophy is a common pathological change in the pathogenesis and progression of multiple cardiovascular diseases, including hypertension, coronary artery disease, and valvular heart disease. Through this pathological process, the heart responds and adapts to stimuli such as ischemia, hypoxia, and pressure or volume overload [1, 2]. Continuous progression of cardiac hypertrophy will eventually result in a decompensated heart and subsequent heart failure [3, 4]. Early reversal of cardiac hypertrophy is an important strategy to postpone heart failure [5].

Healthy adult hearts mainly use long-chain fatty acids and carbohydrates as substrates for energy metabolism. Accumulating studies have shown that the progression of cardiac hypertrophy and failure is accompanied by metabolic changes, including changes in carbohydrate metabolism [6–8]. Schnelle et al. [9] studied glucose carbon metabolism in pressure-overload cardiac hypertrophy using an in vivo [U-13C] glucose labeling strategy. They found that transverse aortic constriction (TAC)-induced cardiac hypertrophy was characterized by a shift in energy metabolism toward a greater reliance on glycolysis. Ho et al. [10] reported that the contribution of ketones to energy production in TAC hearts increased to 18%. In the case of fatty acids, by using a CD36-knockout mouse model, Nakatani et al. [11] found that deficient long-chain fatty acid transportation resulted in aggravated cardiac hypertrophy and reduced systolic function, suggesting the important role of fatty acids in the maintenance of normal systolic function and the myocardial structure. Moreover, further evidence has shown that improving myocardial metabolism usually improves the clinical manifestations and prognosis of heart failure patients [12]. Therefore, changes in energy metabolism are important in the development of cardiac hypertrophy and dysfunction. However, whether specific nonenergetic small-molecule metabolites might be involved in the early pathogenesis of cardiac hypertrophy is not clear.

Chronic metabolic shifts have been considered to be both a cause and a consequence in the pathogenesis of heart dysfunction [12, 13]. We hypothesized that some specific metabolites might be involved in the early pathogenesis of cardiac hypertrophy. To further elucidate early metabolic changes and potential regulatory mechanisms in cardiac hypertrophy, we established a rat model of pressure-overload cardiac hypertrophy via TAC and explored plasma metabolomic changes and possible metabolic pathways at the early stage of pressure overload–induced cardiomyocyte hypertrophy.

## Methods

### Experimental animals and establishment of the TAC model

Male Sprague–Dawley (SD) rats (6 weeks old, body weight 200 g ± 20 g) were provided by the Experimental Animal Center of Chongqing Medical University and housed in an individually ventilated cage (IVC) facility at the Experimental Animal Center of Chongqing Medical University. The animal study protocol was approved by the Ethics Committee of Animal Welfare at the Medical Centers of Chongqing Medical University (Chongqing, China) and Shenzhen University General Hospital (Guangdong, China). All animal experiments complied with the ARRIVE guidelines and were carried out in accordance with the National Institutes of Health Guide for the Care and Use of Laboratory Animals (NIH Publications No. 8023, revised 1978).

Establishment of the model [14]: after anesthesia induction through intraperitoneal pentobarbital injection (60 mg/kg), the rat was immobilized in the supine position atop a heating pad maintained at 37 °C. Endotracheal intubation was performed, and the following parameters were set: tidal volume: 4–6 ml/200 g; respiratory rate: 70 breaths/min; and inspiratory-to-expiratory time ratio: 1:1. Disinfection and skin preparation were performed in the surgical field. After the skin of the left chest was incised, the pectoralis major and the pectoralis minor were separated via blunt dissection to expose the ribs. A horizontal 1.0-cm incision was made between the 2nd and 3rd ribs close to the left side of the sternum under sterilization, the blood vessels and fasciae were separated, and the thymus was gently moved to expose the aortic arch. The aortic arch was lifted with custom-made curved forceps, and a 2–0 silk suture was placed between the innominate and left carotid arteries. A blunt curved 16-G needle (1.6 mm in diameter) was placed next to the aortic arch, and after ligation of the aorta, the needle was promptly removed, and the thymus was replaced in the thoracic cavity. After confirmation of the absence of bleeding during ligation, the chest was closed and the skin incision was sutured. After approximately 10 min of ventilation, when spontaneous breathing was restored, the rat was extubated and returned to the housing facility for recovery.

Fourteen SD rats were randomly subjected to TAC or sham surgery. The sham group underwent the same surgical procedures as the TAC group but without ligation of the aortic arch. One animal in the sham group died of bleeding during the surgery. One animal in the TAC group died of pneumothorax during surgery. Finally, 6 animals in the sham group (n = 6) and 6 animals in the TAC group (n = 6) survived and were used for the experiment. Heart rate and blood pressure were assessed 3 weeks postoperatively by measuring parameters such as blood flow, blood pressure, and pulse at the base of the tail using a rat tail-cuff blood pressure system.

### Echocardiography

Echocardiography was performed 3 weekspostoperatively by technicians from the Department of Sonography who were experienced with sonography of small animals using the Vevo 2100 high-resolution ultrasound imaging system for small animals (Visual Sonics, Canada). After anesthesia induction through intraperitoneal injection of 2% pentobarbital, all limbs of the rat were immobilized on a Styrofoam board, fur on the chest was shaved to fully expose the positions of sternum and left thoracic cage, and M-mode echocardiography was carried out to measure parameters such as diastolic interventricular septal thickness, systolic interventricular septal thickness, end-diastolic left ventricular internal dimension, end-systolic left ventricular internal dimension, diastolic left ventricular posterior wall thickness (LVPWd), systolic left ventricular posterior wall thickness (LVPWs), left ventricular ejection fraction (LVEF), left ventricular fractional shortening (LVFS), left ventricular mass (LV Mass), and left ventricular end-diastolic volume (LVEDV).

### Pathology

All animals were anesthetized using an intraperitoneal injection of 30 mg/kg sodium pentobarbital and then euthanized by thoracotomy and heart removal 3 weeks after surgery. Cardiac tissues were excised and placed in glass receptacles containing phosphate-buffered saline (PBS). Residual blood in the heart was extruded with gentle pressure. After the cardiac tissues were washed with PBS, the residual tissues of the aortic arch and pericardium were carefully removed. With ophthalmology scissors, the atria were removed along the atrioventricular groove, and the right ventricular free wall was cut from the lower right side of the interventricular septum. Thus, the left ventricle and interventricular septum remained. The tissues were fixed in 4% paraformaldehyde for 24 h, embedded in paraffin, and sectioned at 4 µm. Hematoxylin/eosin staining was then used to visualize global morphology. Masson staining was used to assess the fibrosis level. To examine cell size, wheat germ agglutinin (WGA) staining was performed. Slides were stained for 30 min with WGA-FITC-labeled antibody (1:40, L4895, Sigma Aldrich) at room temperature. Staining with Hoechst (1:10,000. 33342, Thermo Scientific) for 5 min was done to visualize the nuclei in blue. Images of tissue sections were captured with a BX53 microscope (Olympus), and analysis was performed in a blinded fashion using ImageJ.

### Untargeted metabolomics analysis

#### Sample collection

The rats were fasted overnight. Then, a blood-drawing needle was used to collect 5 ml of fasting blood from the abdominal aorta of the rats, and the blood was placed in a heparin anticoagulant tube. After sitting at room temperature for 30 min, the blood was centrifuged at 2000 rpm for 10 min and the supernatant was collected, flash-frozen in liquid nitrogen, and stored at − 80 °C for future use.

#### Sample pretreatment

We added 400 µl of prechilled methanol–acetonitrile solution (1:1, v/v) into 100 µl of each sample, and the solution was vortexed for 60 s. The sample was placed at -20 °C for 1 h to precipitate the proteins and centrifuged at 14,000 rcf for 20 min at 4 °C, and the supernatant was collected and lyophilized.

#### Chromatography conditions

An Agilent 1290 Infinity LC ultrahigh-performance liquid chromatography (UHPLC) system and a hydrophilic interaction liquid chromatography (HILIC) column were used for sample separation, with a column temperature of 25 °C and a flow rate of 0.3 ml/min. Mobile phase component A was water + 25 mM ammonium acetate + 25 mM ammonia, and mobile phase B was acetonitrile. Gradient elution procedures were as follows: 0–0.5 min, 95% B; 0.5–7 min, linear transition of B from 95 to 65%; 7–8 min, linear transition of B to 40%; 8–9 min, B maintained at 40%; 9–9.1 min, linear transition of B from 40 to 95%; and 9.1–12 min, B maintained at 95%.

#### Quadrupole time-of-flight (Q-TOF) mass spectrometry (MS) conditions

Testing was carried out using electrospray ionization (ESI) in positive- and negative-ion modes. After UHPLC separation, the sample was subjected to mass spectrometry analysis in an Agilent 6550 mass spectrometer. ESI source parameters were as follows: gas temperature: 250 °C; drying gas: 16 L/min; nebulizer: 20 psig; sheath gas temperature: 400 °C; sheath gas flow: 12 L/min; Vcap: 3000 V; nozzle voltage: 0 V; fragment: 175 V; mass range: 50–1200; acquisition rate: 4 Hz; cycle time: 250 ms.

After testing, an AB Triple TOF 6600 mass spectrometer was used to identify metabolites and collect primary and secondary spectra of quality control (QC) samples. ESI source parameters were as follows: ion source gas 1 (Gas1): 40; ion source gas 2 (Gas2): 80; curtain gas (CUR): 30; source temperature: 650 °C; ion spray voltage floating: ± 5000 V (positive- and negative-ion modes). Secondary spectra were obtained using information-dependent acquisition (IDA) (settings: exclude isotopes within 4 Da; candidate ions per cycle: 10); high-sensitivity mode; declustering potential: ± 60 V (positive- and negative-ion modes); and collision energy: 35 ± 15 eV.

### Statistical analysis

To expand the collection rate of secondary spectra, Q-TOF data collection was segmented according to the following mass ranges: 50–300, 290–600, 590–900, and 890–1200. Four replicates were collected for each method in each segment. The original data collected were transformed into ProteoWizard into.mzXML format, and then peak alignment, retention time correction, and peak area extraction were performed with the XCMS program. Metabolite structures were identified by matching the exact mass (< 25 ppm) and the secondary spectrum against molecules in the database established in our laboratory.

After pretreatment of data obtained from Q-TOFMS and UHPLC-QTOFMS via Pareto-scaling, multidimensional statistical analysis was performed, including unsupervised principal component analysis (PCA), supervised partial least squares discriminant analysis (PLS-DA), and orthogonal partial least squares discriminant analysis (OPLS-DA). Differential metabolites with variable importance in projection (VIP) values > 1.0 in multidimensional statistical analysis were further screened with the unpaired t test (P < 0.05). Finally, metabolic pathway enrichment analysis was performed on the differential metabolites using the Kyoto Encyclopedia of Genes and Genomes (KEGG) database (https://www.genome.jp/kegg/), and receiver operating characteristic (ROC) curve analysis was performed on positive metabolites from the screen to calculate the area under the ROC curve (AUC) and the sensitivity and specificity of the model.

The data are expressed as the mean ± SE, and were statistically analyzed with SPSS software (IBM Corp., version 19.0). Pairwise comparisons were carried out using the unpaired t test. Differences with P < 0.05 were considered statistically significant.

## Results

### Establishment of the cardiac hypertrophy model

**Fig. 1:**
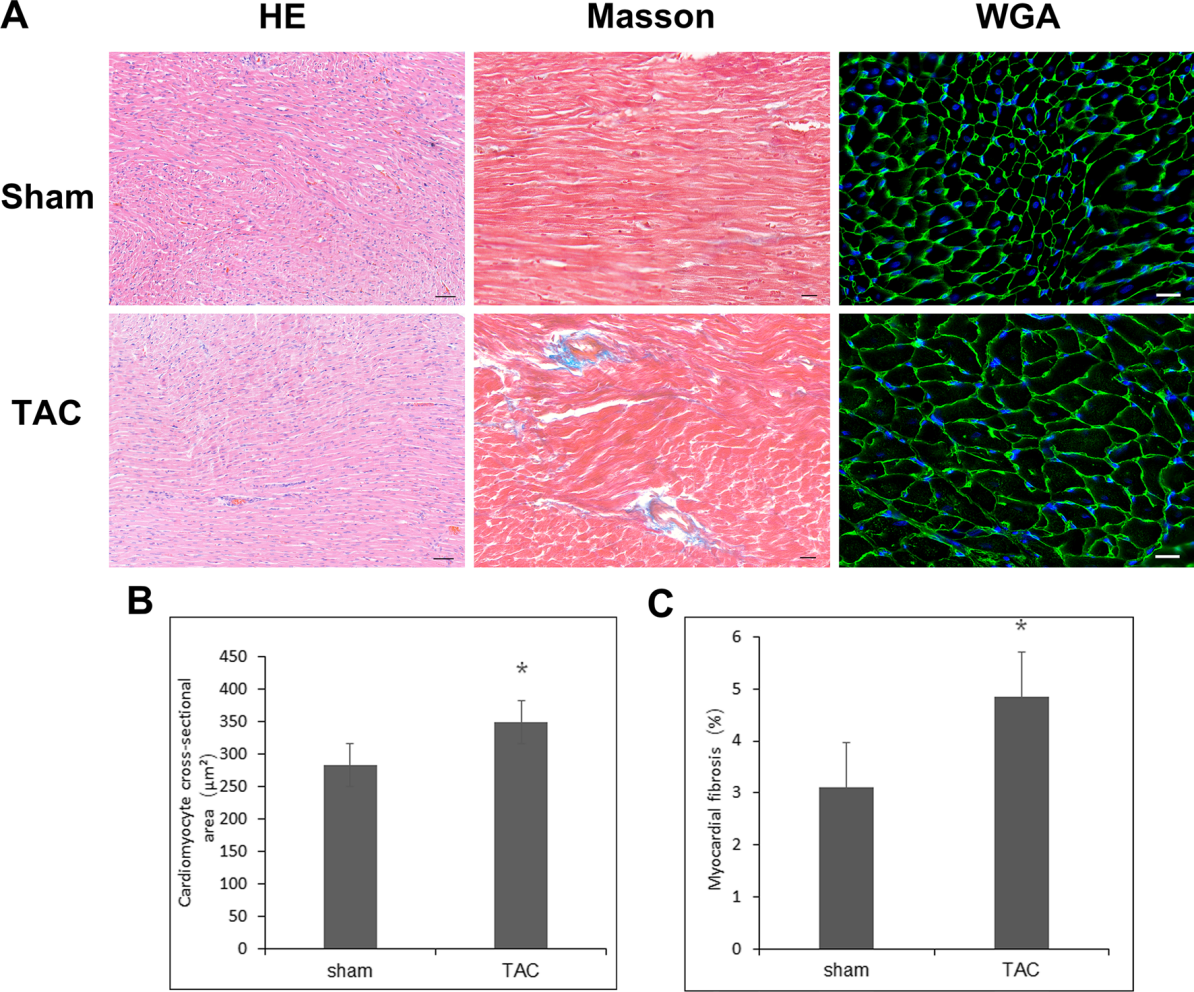
The effects of aortic constriction on cardiomyocyte hypertrophy and fibrosis. **A** Representative images of the left ventricle and the interventricular septum including HE, Masson and WGA staining 3 weeks after TAC surgery. **B** Cardiomyocyte cross-sectional area. **C** Myocardial fibrosis level. *P < 0.05. Bar for HE: 50 µm; Bar for Masson and WGA stains: 20 µm. *HE* Hematoxylin/eosin, *WGA* wheat germ agglutinin, *TAC* transverse aortic constriction

Compared with rats in the sham group, rats in the TAC group had higher tail arterial pressure (158 ± 16.30 mmHg vs 132 ± 5.85 mmHg, n = 6, P < 0.01) 3 weeks after aortic constriction, while no significant difference was observed in heart rate or body weight. Echocardiography suggested trends of greater left ventricular mass, LVPW, and LVEF in the TAC group, but the differences were not significant. Pathological analysis indicated a greater cardiomyocyte cross-sectional area (348.60 ± 9.70 µm^2^ vs 283.20 ± 6.80 µm^2^, n = 6, P < 0.05) and fibrosis level (4.84 ± 0.21% vs 3.11 ± 0.15, n = 6, P < 0.05) in the TAC group than in the sham group. The combined echocardiography and pathology results revealed marked increases in systemic arterial pressure and hypertrophy of cardiomyocytes in rats from the TAC group 3 weeks after surgery, without detectable abnormalities in cardiac structure or function on sonography, indicating early-stage subclinical compensated cardiomyocyte hypertrophy in the rats (Table 1, Fig. 1).

**Table 1:** Changes in blood pressure and cardiac function at 3 weeks after TAC surgery

	Sham (n = 6)	TAC (n = 6)
Body weight (g)	258 ± 18	274 ± 5
Heart beat (BPM)	385 ± 8	407 ± 3
Blood pressure (mmHg)	132 ± 5.85	158 ± 16.30**
IVSd (mm)	1.41 ± 0.11	1.71 ± 0.07
LVPWd (mm)	1.54 ± 0.28	1.77 ± 0.41
LVEDV (ul)	161.38 ± 28.78	203.04 ± 14.94
LVEF (%)	82.91 ± 7.79	86.57 ± 4.31
LV mass (mg)	502.81 ± 38.78	679.44 ± 29.28

Values are mean ± SE. *TAC* transverse aortic constriction, *IVSd* diastolic thickness of intraventricular septum, *LVPWd* left ventricular diastolic posterior wall thickness, *LVEDV* left ventricular end-diastolic volume, *LVEF* left ventricular ejection fraction, *LV mass* left ventricular mass. **P < 0.01

### Metabolic pattern analysis

**Fig. 2:**
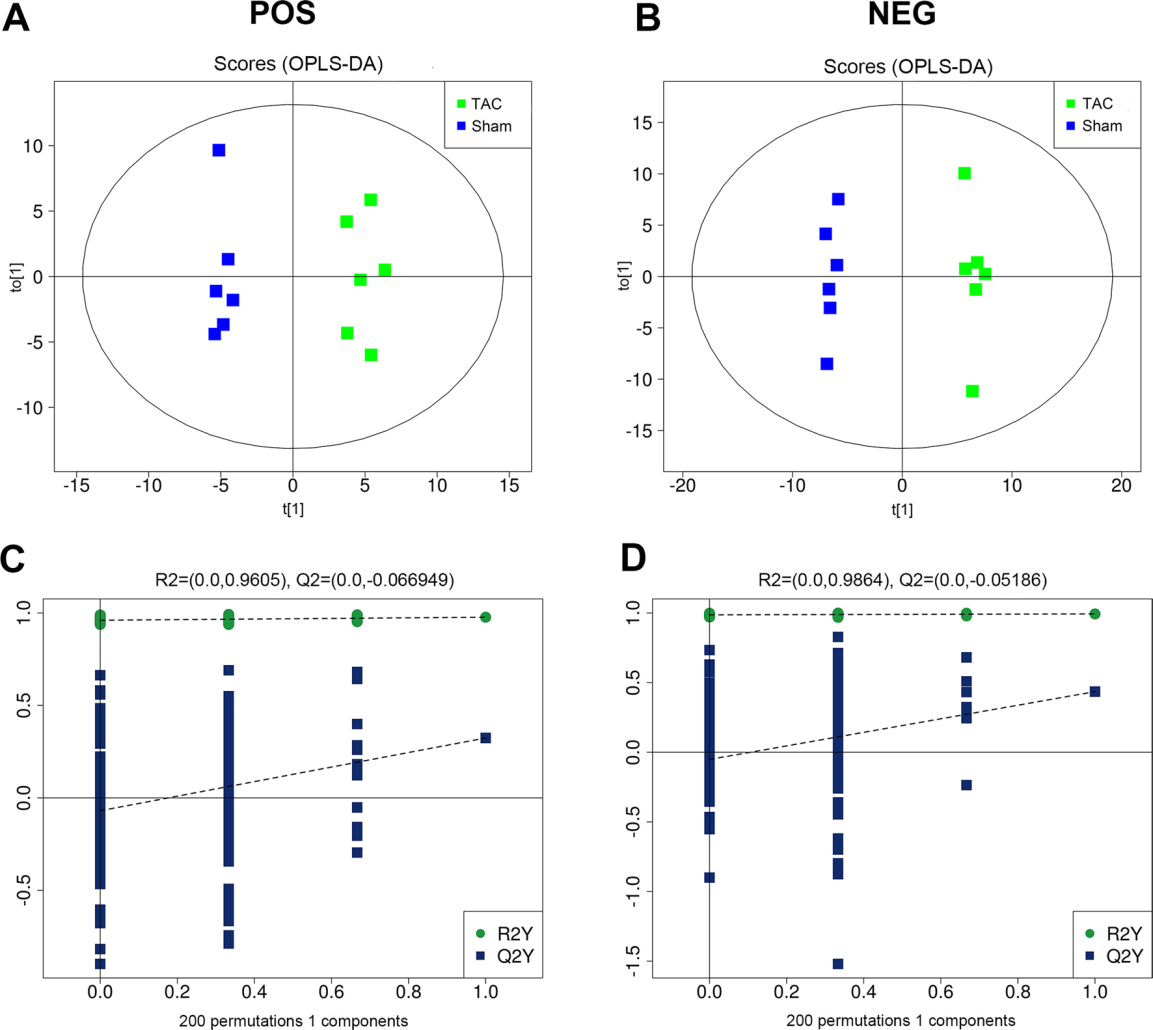
Metabolic pattern analysis by OPLS-DA score plot. **A** and **B** OPLS-DA score plot under positive- (**A**) and negative-ion modes (**B**). Blue circles indicate samples from the sham group (n = 6), and green circles indicate samples from the TAC group (n = 6). **C** and **D** Validated model plots obtained by permutation test: positive- (**C**) and negative-ion mode (**D**). OPLS-DA of the two-dimensional distribution patterns of the metabolites showed that in both positive- and negative-ion modes, significant separation of metabolite patterns was evident when the TAC group was compared with the sham group. The R^2^ and Q^2^ were 0.977 and 0.324 in positive modeand 0.993 and 0.435 in negative mode, respectively. OPLS-DA: orthogonal partial least squares discriminant analysis

Comparison of the total ion chromatogram between samples from the TAC and sham groups in positive- and negative-ion modes revealed that the intensities and retention times of the peaks mostly overlapped, indicating that the variations caused by instrument errors were relatively small throughout the experimental process. OPLS-DA of the two-dimensional distribution patterns of the metabolites showed that in both positive- and negative-ion modes, significant separation of metabolite patterns was evident when the TAC group was compared with the sham group. The R^2^ and Q^2^ were 0.977 and 0.324 in positive mode and 0.993 and 0.435 in negative mode, respectively. The results indicated good data fitting and reproducibility, a stable and effective model, and metabolite patterns that distinguished the sham group from the group with early-stage compensated cardiomyocyte hypertrophy (Fig. 2).

### Differential metabolites

**Fig. 3:**
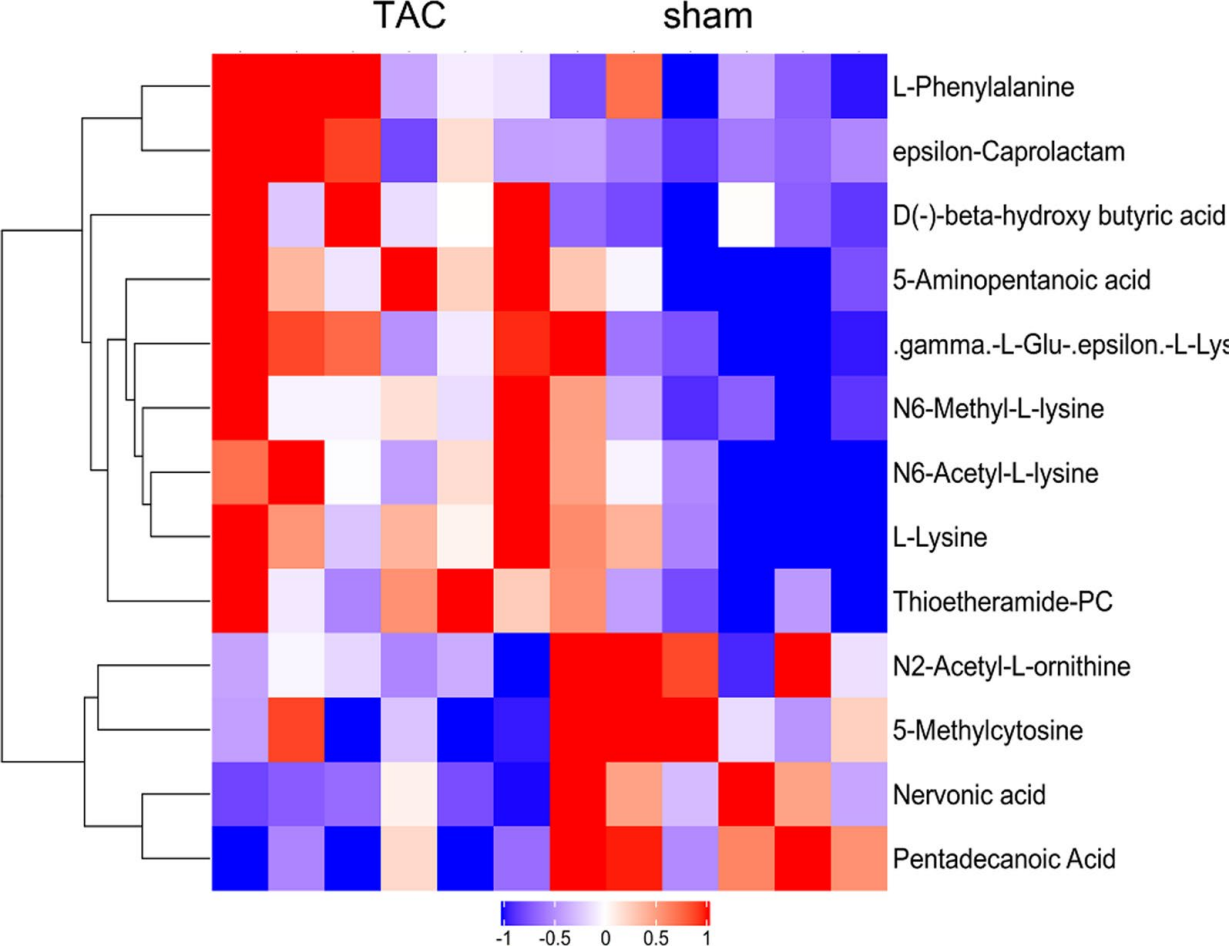
Unsupervised hierarchical clustering heat map of metabolites obtained from the plasma of rats after TAC surgery.* TAC* transverse aortic constriction

We analyzed differential metabolites in depth between the sham group and TAC group 3 weeks after surgery. Based on the VIP values of characteristic variables obtained from the OPLS-DA model, the differential metabolites with VIP > 1 and p < 0.05 were identified. Thirteen metabolites showed significant differences between the groups at 3 weeks postoperatively (Table 2, Fig. 3), including amino acids and polypeptides (5-aminopentanoic acid, *N*6-acetyl-l-lysine, l-lysine, *N*6-methyl-l-lysine, *N*2-acetyle-l-ornithine, l-phenylalanine), fatty acids (pentadecanoic acid, nervonic acid), and pyrimidine (5-methylcytosine).

**Table 2:** Significantly different metabolites identified 3 weeks after TAC surgery

Name of metabolites	Category	VIP	FC	p
5-Aminopentanoic acid	Amino acids, peptides	1.13	1.45	0.004
*N*6-Acetyl-l-lysine	Amino acids, peptides	1.21	1.46	0.023
l-Lysine	Amino acids, peptides	9.94	1.30	0.028
ε-Caprolactam	Others	2.91	1.40	0.029
γ-l-Glu-ε-l-lysine	Others	2.96	1.45	0.030
*N*6-Methyl-l-lysine	Amino acids, peptides	2.17	1.34	0.033
*N*2-Acetyl-l-ornithine	Amino acids, peptides	1.12	0.78	0.034
Thioetheramide-PC	Others	1.12	1.40	0.036
5-Methylcytosine	Pyrimidines	2.72	0.82	0.037
Pentadecanoic Acid	Fatty acids	3.08	0.50	0.003
d(−)-β-Hydroxybutyric acid	Others	2.11	2.31	0.008
Nervonic acid	Fatty acids	2.05	0.44	0.018
l-Phenylalanine	Amino acids, peptides	2.47	1.41	0.030

TAC: transverse aortic constriction; VIP: variable importance in projection; FC: fold change

### KEGG pathway analysis

**Fig. 4:**
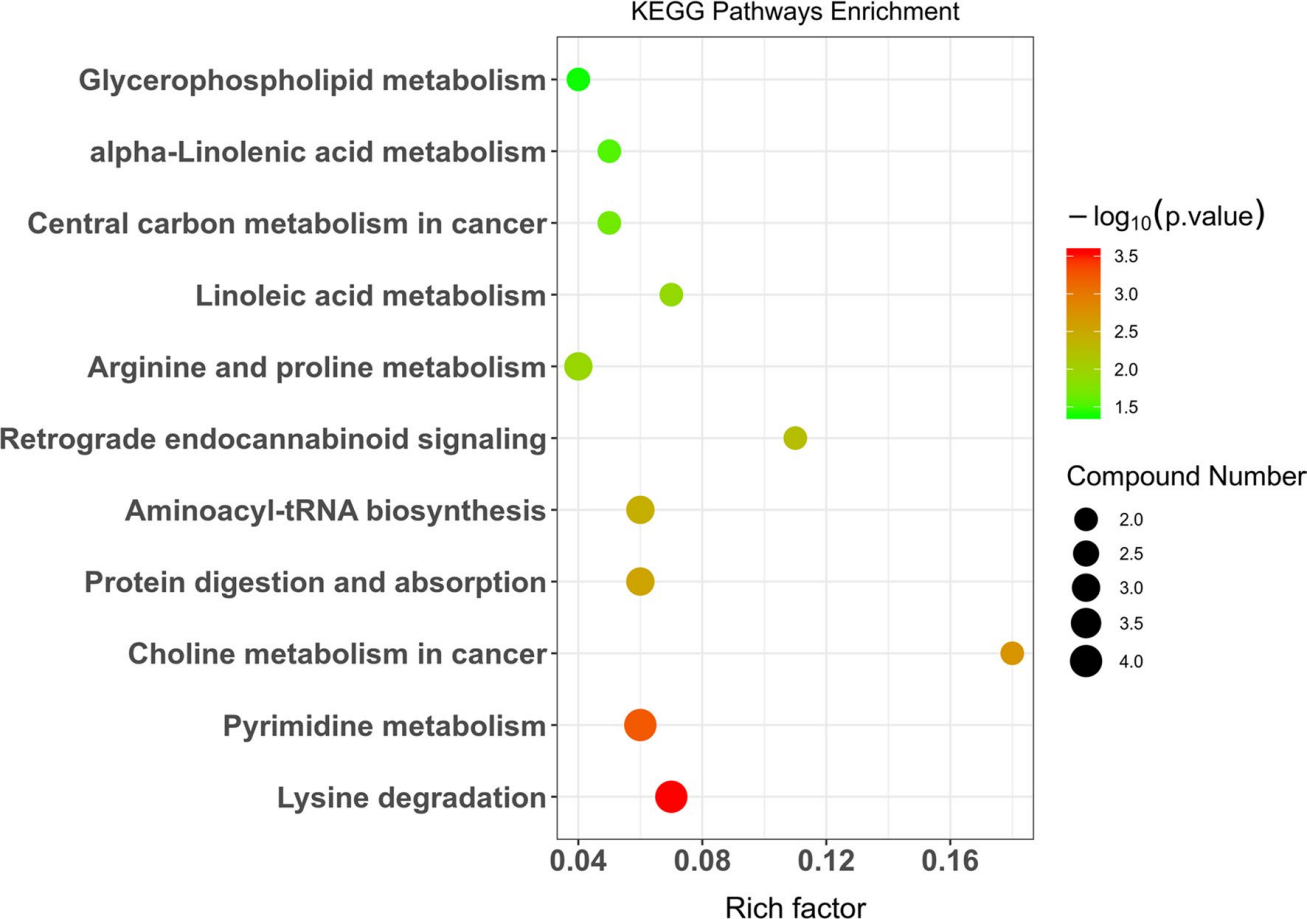
Enriched KEGG pathways between the sham and TAC groups presented as a bubble diagram based on significantly different metabolites

We analyzed metabolic pathway changes in the TAC group 3 weeks postoperatively compared with the sham group and found that the metabolic pathways involved in early-stage cardiomyocyte hypertrophy included lysine degradation, pyrimidine metabolism, aminoacyl-tRNA biosynthesis, arginine and proline metabolism, linoleic acid metabolism, central carbon metabolism, and glycerophospholipid metabolism. The lysine degradation pathway exhibited the most marked changes. The metabolites related to lysine degradation were 5-aminopentanoic acid, N6-acetyl-l-lysine, and l-lysine (Table 3, Fig. 4).

**Table 3:** The significantly altered pathways between TAC and sham rats

Pathway	Metabolites	P value
Lysine degradation	5-Aminopentanoic acid, N6-acetyl-l-lysine, l-lysine	0.0002
Pyrimidine metabolism	Thymidine, pseudouridine,5-Methylcytosine, thymine	0.0005
Choline metabolism in cancer	PC(16:0/16:0), SOPC	0.0019
Protein digestion and absorption	l-Phenylalanine, l-Lysine, l-Histidine	0.0027
Aminoacyl-tRNA biosynthesis	l-Phenylalanine, l-lysine, l-histidine	0.0037
Retrograde endocannabinoid signaling	PC(16:0/16:0), SOPC	0.0058
Arginine and proline metabolism	5-Aminopentanoic acid, 4-Guanidinobutyric acid, D-proline	0.0115
Linoleic acid metabolism	PC(16:0/16:0), SOPC	0.0125
Central carbon metabolism in cancer	l-Phenylalanine, l-histidine	0.0214
alpha-Linolenic acid metabolism	PC(16:0/16:0), SOPC	0.0296
Glycerophospholipid metabolism	PC(16:0/16:0), SOPC	0.0403

SOPC: 1-stearoyl-2-oleoyl-sn-glycerol 3-phosphocholine

### ROC analysis

**Fig. 5:**
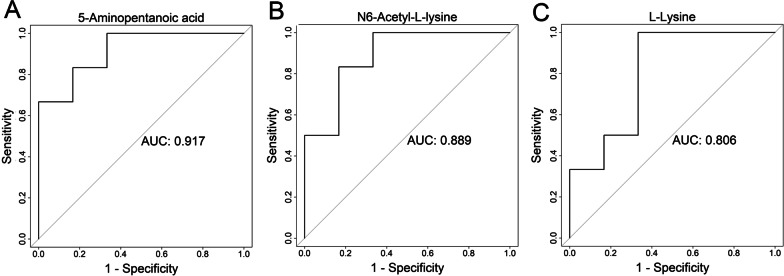
ROC analysis of metabolites associated with the lysine degradation pathway

We next calculated the predictive value of the three metabolites related to the lysine degradation pathway for early-stage cardiomyocyte hypertrophy. The AUC values of 5-aminopentanoic acid, N6-acetyl-l-lysine, and l-lysine for predicting compensated cardiomyocyte hypertrophy were 0.917, 0.889, and 0.806, respectively (Fig. 5).

## Discussion

Cardiac hypertrophy is a common pathological characteristic during the pathogenesis and development of multiple cardiovascular diseases, such as hypertension, coronary artery disease, and valvular heart disease, and early reversal of cardiac hypertrophy has great value in maintaining heart function and delaying heart failure [14, 15]. In this study, we established a cardiac hypertrophy model through TAC and found pathological evidence of cardiomyocyte hypertrophy and fibrosis 3 weeks after surgery, although no significant increase in ventricular wall thickness or decrease in cardiac function was detectable through echocardiography, indicating that pathological changes may occur during early-stage subclinical cardiomyocyte hypertrophy when clinical indications of risk factors for cardiac remodeling are present but no thickening of the ventricular wall is detectable on sonography. In fact, pathological cardiac hypertrophy is difficult to reverse once formed [16]. In addition, for patients with risk factors for cardiac hypertrophy, in addition to actively controlling the risk factors, early detection and intervention of cardiomyocyte hypertrophy are of great importance to the protection of the target organ and the prevention and treatment of chronic heart failure.

Heart failure is usually accompanied by changes in the energy metabolism of cardiomyocytes. Cardiomyocyte metabolism often changes before cardiac structure [17, 18]. However, whether metabolic changes can allow early detection of subclinical cardiomyocyte hypertrophy is still unclear. We studied early-stage cardiomyocyte hypertrophy 3 weeks after TAC surgery, analyzed the plasma metabolomic changes through UHPLC-QTOFMS, and uncovered significant separation of metabolic patterns between the sham and TAC groups, indicating that the two-dimensional distribution patterns of plasma metabolites can be used to identify early-stage cardiomyocyte hypertrophy. Furthermore, we screened for differential metabolites by setting the thresholds of VIP > 1 and P < 0.05, according to the VIP values of characteristic variables obtained from the OPLS-DA model, and found 13 metabolites that showed significant differences between groups at 3 weeks postoperatively, including amino acids and polypeptides such as 5-aminopentanoic acid, *N*6-acetyl-l-lysine, l-lysine, *N*6-methyl-l-lysine, *N*2-acetyl-l-ornithine, and l-phenylalanine; fatty acids such as pentadecanoic acid and nervonic acid; and pyrimidines such as 5-methylcytosine. Among these metabolites, the levels of 5-aminopentanoic acid, *N*6-acetyl-l-lysine, l-lysine, l-phenylalanine, and thymidine were increased, while the levels of *N*2-acetyl-l-ornithine, 5-methylcytosine, and pentadecanoic acid were decreased. Consistent with Sansbury et al. [19], we found that the amino acid changes caused by cardiac hypertrophy were the most pronounced. However, Sansbury et al. [19] reported more significant changes in branched-chain amino acids during cardiac hypertrophy and heart failure. Branched-chain amino acids are usually related to metabolic factors such as insulin resistance. We found that changes in amino acid metabolism, mainly lysine metabolism, were more pronounced during early cardiomyocyte hypertrophy. Lysine is an essential amino acid for humans and mammals. Because its concentration in cereals and foods is very low and it is prone to destruction during processing, lysine is also called the first-limiting amino acid. Lysine has important functions in the promotion of human physiologic development and fatty acids oxidation. Fust et al. [20] found that the addition of lysine to the diet could treat osteoporosis. Shimomura et al. [21] found that moderate dietary supplementation with lysine could relieve vascular calcification in uremic rats, while the plasma lysine content was not increased by the addition of lysine to the diet. Our study found that at the early stage of subclinical cardiomyocyte hypertrophy caused by pressure overload, plasma *N*6-acetyl-l-lysine, l-lysine, and *N*6-methyl-l-lysine were all elevated, suggesting that changes in metabolites of amino acids such as lysine may become early predictive plasma markers for subclinical cardiomyocyte hypertrophy caused by pressure overload.

KEGG pathway enrichment analysis uncovered the metabolic pathways involved in cardiomyocyte hypertrophy at 3 weeks, including lysine degradation, pyrimidine metabolism, aminoacyl-tRNA biosynthesis, arginine and proline metabolism, linoleic acid metabolism, central carbon metabolism, and glycerophospholipid metabolism. The lysine degradation pathway exhibited the most pronounced changes. Posttranslational modifications of proteins have important functions in the growth, differentiation, and metabolic regulation of cells. Dueto breakthroughs in detection techniques, research on protein phosphorylation has progressed very quickly, and many reports on the involvement of signal molecule phosphorylation in cardiac hypertrophy are available [22, 23]. With advancements in the techniques of high-resolution mass spectrometry-based omics, the understanding of the epigenetic modifications of histones has progressed far in recent years [3, 24]. Among the proteins expressed by mammals, more than 50% can have various posttranslational modifications at certain times and in certain subcellular locations, which is a way the body precisely regulates pathological and physiological processes. Such posttranslational modifications are mainly reversible modifications of certain amino acid residues, and lysine is one of the most frequently modified residues [25].

The effects of methylation, acetylation, ubiquitination, and glycosylation of lysine on cardiovascular diseases have garnered much attention [26]. With continuous improvements in the sensitivity, scan speed, and resolution of biological mass spectrometry, an increasing number of acylation modifications of lysine have been discovered, such as succinylation and malonylation. The lysine modifications of histones undergo dynamic changes under the effects of regulatory enzymes and exert regulatory functions on gene transcription by altering the interaction between histones and DNA and recruitment of binding proteins [27].

Metabolomics can provide new clues on the pathogenic mechanisms, severity, progression, and potential treatment methods of disease through the measurement of target metabolites [28]. In this study, we found that at the early stage of pressure overload-induced cardiomyocyte hypertrophy, the levels of metabolites related to lysine degradation, such as 5-aminopentanoic acid, *N*6-acetyl-l-lysine, and l-lysine, were all elevated in the TAC group compared with the sham group, indicating that acetylation of lysine may be involved in the pathogenesis of pressure overload–induced early-stage cardiomyocyte hypertrophy. The elevated lysine degradation might be a response of the heart to pressure overload by TAC. Since lysine modification can exert regulatory functions on gene transcription, the elevation of lysine degradation-related metabolites might also be a cause of cardiomyocyte hypertrophy. The ROC AUC values of 5-aminopentanoic acid, *N*6-acetyl-l-lysine, and l-lysine for predicting compensated cardiomyocyte hypertrophy were 0.917, 0.889, and 0.806, respectively, indicating that 5-aminopentanoic acid, *N*6-acetyl-l-lysine, and l-lysine may become plasma markers for the prediction of pressureoverload-induced early-stage subclinical cardiomyocyte hypertrophy. However, the results of this study mainly came from animals, with the limitations inherent to them. Therefore, the relationships between lysine modifications and the clinical importance of such relationships await further verification.

## Conclusion

Cardiac hypertrophy accompanies nonenergetic metabolism. Disruption of lysine degradation might be involved in the early pathology of cardiomyocyte hypertrophy, and related metabolites, including 5-aminopentanoic acid, *N*6-acetyl-l-lysine, and l-lysine, might be potential predictive and interventional targets for subclinical cardiomyocyte hypertrophy.

## Data Availability

The datasets generated and/or analyzed during the current study are not publicly available due to privacy or ethical restrictions, but are available from the corresponding author upon reasonable request.

## Abbreviations

HILIC: Hydrophilic interaction liquid chromatography
IVSd: Diastolic thickness of the intraventricular septum
KEGG: Kyoto encyclopedia of genes and genomes
LVPWd: Left ventricular diastolic posterior wall thickness
LVEDV: Left ventricular end-diastolic volume
LVEF: Left ventricular ejection fraction
LV mass: Left ventricular mass
MS: Mass spectrometry
OPLS-DA: Orthogonal partial least squares discriminant analysis
Q-TOF: Quadrupole time-of-flight
ROC: Receiver operating characteristic
SOPC: 1-Stearoyl-2-oleoyl-sn-glycerol 3-phosphocholine
TAC: Transverse aortic constriction
UHPLC: LC ultrahigh-performance liquid chromatography
VIP: Variable importance in projection
WGA: Wheat germ agglutinin
